# Research on the treatment effects and drug resistances of long-term second-line antiretroviral therapy among HIV-infected patients from Henan Province in China

**DOI:** 10.1186/s12879-018-3489-7

**Published:** 2018-11-15

**Authors:** Junli Chen, Min Zhang, Mingquan Shang, Weiwei Yang, Zhe Wang, Hong Shang

**Affiliations:** 1 NHC Key Laboratory of AIDS Immunology (China Medical University), Department of Laboratory Medicine, The First Affiliated Hospital of China Medical University, No 155, Nanjing North Street, Heping District, Shenyang, 110001 Liaoning Province China; 2 Key Laboratory of AIDS Immunology of Liaoning Province, The First Affiliated Hospital of China Medical University, Shenyang, 110001 China; 3 Key Laboratory of AIDS Immunology, Chinese Academy of Medical Sciences, Shenyang, 110001 China; 4 Collaborative Innovation Center for Diagnosis and Treatment of Infectious Diseases, 79 Qingchun Street, Hangzhou, 310003 China; 5 Disease Prevention and Control Center of Henan Province, Zhengzhou, 450016 China

**Keywords:** HIV-1, HAART, Second-line antiretroviral therapy, CD4 count, Viral load, Drug resistance

## Abstract

**Background:**

HIV/AIDS patients who fail to respond to first-line treatment protocols are switched to second-line ART. Identifying factors that influence effective second-line treatment can improve utilization of limited medical resources. We investigated the efficacy of long-term second-line anti-retroviral therapy (ART) after first-line virologic failure as well as the impact of non-nucleotide reverse transcriptase inhibitor (NNRTI), nucleotide reverse transcriptase inhibitor (NRTI), and protease inhibitor (PI) resistance mutations and medication adherence on ineffective viral suppression.

**Methods:**

A total of 120 patients were evaluated at 6, 12, 18, 24, and 48 months after initiation of second-line ART; a paper questionnaire was administered via a face-to-face interview and venous blood samples were collected. CD4^+^ T cell count, viral load, and drug resistance genotypes were quantified.

**Results:**

CD4^+^ T cell counts increased from 170 cells/μL (IQR 100–272) at baseline to 359 cells/μL (IQR 236–501) after 48 months of second-line treatment. Viral load (log_10_) decreased from 4.58 copies/mL (IQR 3.96–5.17) to 1.00 copies/mL (IQR 1.00–3.15). After switching to second-line ART, nine patients newly acquired the NRTI drug-resistant mutation, M184 V/I. No major PI resistance mutations were detected. Logistical regression analysis indicated that medication adherence < 90% in the previous month was associated with ineffective viral suppression; baseline high/low/moderate level resistance to 3TC/TDF was protective towards effective viral suppression.

**Conclusions:**

Long-term second line ART was effective in the Henan region of China. Drug resistance mutations to NRTIs were detected in patients receiving second-line ART, suggesting that drug resistance surveillance should be continued to prevent the spread of resistant strains. Patient medication adherence supervision and management should be strengthened to improve the efficacy of antiviral treatment.

## Background

In 2003, the “Four Free and One Care” antiretroviral therapy treatment (ART) policy was formally implemented in China [1]. This policy is comprised of 1) free antiretroviral therapy to rural patients and low-income urban patients, 2) free HIV screening and counseling, 3) free ART for pregnant women with HIV to prevent mother-to-child transmission along with free HIV testing for newborns, 4) free education for children orphaned by AIDS, and free care and economic assistance to households with members suffering from HIV/AIDS [2]. In accordance with these guidelines, the first line therapy regimen for HIV patients includes two nucleotide analogue reverse transcriptase inhibitors (NRTIs) and one non-nucleotide analogue reverse transcriptase inhibitors (NNRTIs) [3]. However, a proportion of patients who have initiated first-line ART subsequently develop drug resistance, which has been growing as the duration of ART treatment increases in China [4–7]. HIV drug resistance caused by the antiretroviral drug selection pressure is the primary reason for clinical failure of ART. In 2009, patients with failure of the first-line treatment protocol have switched to second-line ART, which is comprised of 3TC (lamivudine), TDF (Tenofovir), and Lpv/r (Lopinavir/ritonavir). More than 55,000 patients have received second line ART in China as of 2016 [8].

Although second-line treatment regimens include two NRTIs, 3TC and TDF, multidrug resistance and cross-resistance mutations acquired during first-line ART often results in only partial effectiveness of the NRTIs during second-line treatment. Some small sample size studies conducted in China have reported an increase in CD4^+^ T cells in HIV-infected patients within 12 months of the switch to second-line treatment; HIV viral loads were lower than at the time of first-line failure in these patients as well [9–11]. Ding et al’s found that viral suppression rates reached 96.3% (viral load < 400 copies/ml) and CD4^+^ T cell counts were 531 after 36 months of second-line ART in patients from northeast China [12]. However, medical resources are unequal among different regions and AIDS-treatment institutions in China [13]; thus, long-term studies of the effectiveness of second-line treatment are still necessary in China. Investigation of the efficacy of second-line ART in resource-limited countries has revealed an increase in viral suppression and CD4^+^ T cell counts within 24 months of switching to second-line therapy [14–17]. The EARNEST trial in sub-Saharan African countries showed that antiviral therapy was effective during the long-term second-line therapy [18]. However, viral subtypes and composition of the first-line protocol often differ between China and other countries. Thus, long-term observational study of second-line treatment efficacy in China is needed.

Drug selection pressure caused by the therapy regimen changes can lead to the accumulation of drug resistance mutations during long-term treatment. An increase in NRTI drug resistance was reported in patients after switching to the second line regimen [19]. Protease inhibitors (PI) such as Lpv/r (Lopinavir/ritonavir) replace NNRTIs in second-line regimens as it has been reported that mutations resulting in lopinavir resistance are infrequent [20–22]. However, Olawale Ajose et al. reported that PI resistance mutations were found in 18% of patients as determined via HIV genotyping [23]. In order to effectively guide treatment, it is essential to know whether different resistance mutations appear during long term second-line ART.

Here, we describe a prospective study of a cohort of patients with AIDS within 4 years of initiation of second-line treatment in Weishi County of Henan Province in China. We examined both the effectiveness of second-line treatment as well as associated factors which can impact treatment; importantly, we also analyzed whether cumulative NRTI resistance mutations acquired during first-line therapy failure affected the efficacy of second-line therapy. We also analyzed changes in PI, NRTI, and NNRTI resistance mutations after long-term second-line therapy.

## Methods

### Ethics statement

This research was approved by the Institutional Review Board of the first Affiliated Hospital of China Medical University. All participants signed informed consent statements.

### Study population

Patients were recruited from Henan between August 2009 and February 2010. Inclusion criteria were those listed in the Chinese National Free AIDS Antiretroviral Therapy Manual issued in 2009 [24] and in the WHO guidelines for antiretroviral therapy for HIV infection in adults and adolescents [25]: (1) ≥18 years of age; (2) virologic failure of first-line therapy, defined as a plasma HIV-1 RNA level > 400 copies/mL after 6 months of treatment or an HIV-1 RNA level > 1000 copies/mL after initial virologic suppression; (3) virologic treatment failure confirmed: viral load > 1000 copies/mL after patients resumed first-line treatment for one month with rectified treatment compliance; (4) second-line therapy protocol including lamivudine (3TC), tenofovir (TDF), and lopinavir/ritonavir (LPV/r) as the second-line antiretroviral regimen; and (5) at least one follow-up visit after the switch to the second-line antiretroviral therapy regimen.

After 6, 12, 18, 24, and 48 months of second-line therapy, we administered a paper questionnaire through a face-to-face interview and collected blood samples to assess patients’ CD4^+^ T cell count, HIV viral load, and “in-house” HIV genotype drug resistance test. During second-line therapy, “ineffective viral suppression” is defined as follows: plasma HIV-1 RNA level > 1000 copies/mL after 12 months of treatment; viral rebound which is defined as plasma HIV-1 RNA level > 1000 copies/mL after initial virologic suppression during second-line ART.

### Data acquisition

CD4^+^ T lymphocyte count: a FACSCalibur flow cytometer (Becton Dickinson, USA) was used to enumerate CD4^+^, CD8^+^, and CD3^+^ T lymphocytes. Peripheral venous blood was collected and treated with EDTA-3 K anticoagulant; T lymphocyte counts were obtained within 24 h.

Viral load: Plasma obtained from patients’ venous blood samples was stored at − 80 °C until testing. HIV viral load was determined using a COBAS AmpliPrep/COBAS TaqMan HIV-1 test (Roche, Switzerland). The detection range of the COBAS Assay for viral load quantification is 20–10^7^ copies/mL.

Drug-resistant mutation analysis: Mutations in the HIV polymerase gene, Pol, were screened for drug resistance mutations. *HIV-1* viral RNA was extracted from 200 μl plasma with a QIAamp Viral RNA Mini Kit (Qiagen, Germany). One-step Reverse transcription-polymerase chain reaction (RT-PCR) was carried out using a TaKaRa One-step RNA PCR Kit (Takara Bio, China). The HIV-1 *pol* gene was amplified using first round primers MAW26 (5’-TTGGAAATGTGGAAAGGAAGGAC-3′; HXB2 2028–2050) and RT21 (5’-CTGTATTTCTGCTATTAAGTCTTTTGATGGG-3′; HXB2 3509–3539); amplification was achieved using 1 cycle of 50°C for 30 min, 1 cycle of 94 °C for 5 min, and 30 cycles of 94 °C for 30 s, 55 °C for 30 s, and 72 °C for 2 min 30 s, with a final extension of 72 °C for 10 min in the first round; and second round primers PRO-1 (5’-CAGAGCCAACAGCCCCACCA-3′; HXB2 3509–3539) and RT4R (5’-CTTCTGTATATCATTGACAGTCCAGCT-3′; HXB2 3509–3539);amplification was achieved using 1 cycle of 94 °C for 5 min and 30 cycles of 94 °C for 30 s, 63 °C for 30 s, and 72 °C for 2 min 30 s, with a final extension of 72°Cfor 10 min [11, 26]. Positive, negative, and blank controls were included for PCR quality control; positive control: HIV-positive specimens and containing the *pol* gene; negative control: specimens that are HIV-negative; blank control: amplification without template. The negative and positive controls were extracted, amplified, detected, and analyzed simultaneously with the research sample. Sequences were aligned using Contig software and edited using Bioedit software. The resulting sequences were submitted to the Stanford University HIV drug resistance database (http://hivdb.stanford.edu) for interpretation of putative drug resistance results.

### Statistical analysis

SPSS software (version 17.0) was used to analyze quantitative data. Categorical data was described by rate or ratio and analyzed by either Chi-square test or Fisher’s exact test. Continuous data was described by the mean and standard deviation if data met the hypothesis of normal distribution, otherwise median and inter-quartile ranges (IQRs) were used. Univariate and multivariate logistic regression were performed to identify possible associated factors that may have contributed to viral suppression. *P* < 0.05 for two-sided tests was defined as statistically significant.

## Results

### Baseline information for HIV-infected patients undergoing long-term second-line antiretroviral therapy

Of the 120 patients that met the inclusion criteria for second-line therapy in our cohort, the median age was 45.2 (39.8–52.3) years, 60.8% (73/120) were male, 39.2% were female (47/120). 95.8% (115/120) of patients were infected through paid blood donation(blood transfusion). All HIV Pol sequences were subtype B interpreted by the Stanford University HIV drug resistance database. All 120 patients used first-line therapy regimens (2NRTIs + 1NNRTIs); first-line treatment duration was 5.0 (3.7–5.5) (median, IQR) years. At initiation of second-line therapy, patient baseline viral load (lg10) (median, IQR) and mean CD4^+^ T cell count (median, IQR) were 4.58 (3.96–5.17) copies/ml and 170 (100–272) cells/μl, respectively. After first-line failure, drug resistance rates to 3TC and TDF were 32.5% (38/117) and 48.7% (57/117), respectively (Table 1). None of the patients exhibited resistance to LPV/r.

**Table 1:** Baseline information before second-line antiretroviral treatment

Variable	Category	Numerical value
Gender (n. %)	Male	73(60.8%)
	Female	47(39.2%)
Age (years, IQR)		45.2(39.8–52.3)
Education degree (n, %)	Primary school	61(50.8%)
	Junior middle school	40(33.3%)
	Illiteracy	19(15.8%)
Occupation (n, %)	Farmer	120(100%)
Route of infection (n, %)	Paid blood donation(blood transfusion)	115(95.8%)
	Heterosexual	3(2.5%)
	Blood transfusion	1(0.8%)
	Unknown	1(0.8%)
Duration of first-line treatment (years, IQR)		5.0(3.7–5.5)
NRTIs drug resistance (n, %)		68(58.1%)
	3TC drug resistance	38(32.5%)
	TDF drug resistance	57(48.7%)
NNRTIs drug resistance (n, %)		84(71.8%)
Lpv/r drug resistance (n, %)		0(0)
Baseline viral load (lg10) (median, IQR) copies/ml		4.58 (3.96–5.17)
Baseline CD4 + T cells (median, IQR) cells/μl		170 (100–272)

*3TC* Lamivudine, *TDF* Tenofovir, *NRTIs* Nucleoside reverse transcriptase inhibitor, *NNRTIs* Non-nucleoside reverse transcriptase inhibitor, *Lpv/r* Lopinavir/ritonavir

### Efficacy of long-term second-line antiretroviral treatment

Staff members regularly followed up with patients. After switching to second-line treatment, 102, 104, 82, 90, and 85 patients were retained for follow-up at 6, 12, 18, 24, and 48 months, respectively. Of the 16 patients who were lost to follow-up at 6 months, two continued second-line therapy according to drug receipt records from the CDC; however, we were unable to collect blood samples and therapy records from these two patients as they were away from Weishi county, Henan province at 6 months. We were able to retrieve blood samples and therapy information from these patients at 12 month. The number of patients who were lost to follow-up increased to 30 at 18 months; Another eight patients continued second-line therapy according to drug receipt records from the CDC; however, we were unable to collect blood samples and therapy records from these patients as they were away from Weishi county at the 18 month timepoint. We were able to obtain their blood samples and therapy information at the 24 month timepoint. Finally, 35 patients were lost to follow-up at 48 months.

During long-term second-line therapy, patients’ CD4^+^ T cells increased from a baseline of 170 cells/μL (IQR 100–272) at initiation of second-line therapy to 359 cells/μL (IQR 236–501) after 48 months of treatment, which was statistically significant (*P* < 0.05) (Table 2). The proportion of patients whose CD4^+^ T cells counts were > 350 cells/μL at baseline, 6, 12, 18, 24, and 48 months after initiation of second-line treatment increased (trend test chi-square value = 52.3, < 0.001).

**Table 2:** Results of CD4 + T cells and viral load within the 48 months second-line antiretroviral treatment

Duration of treatment (month)	Patients followed-up (n)	Viral load (lg10) (median, IQR) copies/ml	Viral load compared with baseline	CD4 + T cells (median, IQR) cells/μl	CD4 compared with baseline
Baseline	120	4.58(3.96–5.17)	–	170(100–272)	–
6	102	2.02(1.00–3.84)	*P* < 0.001	230(164–323)	*P* < 0.001
12	104	1.60(1.00–3.38)	*P* < 0.001	246(185–349)	*P* < 0.001
18	82	1.77(1.00–3.87)	*P* < 0.001	296(186–394)	*P* < 0.001
24	90	1.70(1.00–3.90)	*P* < 0.001	316(205–436)	*P* < 0.001
48	85	1.00(1.00–3.15)	*P* < 0.001	359(236–501)	*P* < 0.001

Viral load (log_10_) decreased from an average baseline level of 4.58 copies/mL (IQR 3.96–5.17) to 1.00 copies/mL (IQR 1.00–3.15) after 48 months of second-line therapy. HIV viral load decreased significantly (P < 0.05) after several different durations of second-line treatment (Table 2). The proportion of patients whose viral load was < 1000copies/mL increased with time of second-line treatment (trend test chi-square value = 108.0, *P* < 0.001). The proportion of patients with viral loads < 1000 copies/mL at 6, 12, 18, 24, and 48 months was 61.8% (63/102), 71.1% (74/104), 69.5% (57/82), 70.0% (63/90), and 75.3% (64/85), respectively. The proportion and frequency of patients with < 50 HIV-RNA copies/mL at 6, 12, 18, 24 and 48 months was 43.1% (44/102), 43.4% (45/104), 42.7% (35/82), 50% (45/90), and 67.1% (57/85), respectively.

### PI/NRTI/ NNRTI drug resistance mutations among patients with virologic treatment failure during second-line treatment

In-house genotype drug resistance tests were performed on samples from patients whose viral loads were > 1000 copies/ml. All sequences obtained during **s**econd-line treatment were HIV subtype B as interpreted by the Stanford University HIV drug resistance database. Rates of drug resistance to NRTIs were 58.1% (68/117), 43.3% (13/30), 31.8% (7/22), 28.0% (7/25), 18.1% (2/11), 31.6% (6/19) at baseline, 6, 12, 18, 24, and 48 months, respectively. Rates of drug resistance rates to NNRTIs were 71.8% (84/117), 56.7% (17/30), 45.5% (10/22), 24.0% (6/25), 36.3% (4/11), and 36.8% (7/19) at baseline, 6, 12, 18, 24, and 48 months, respectively. No major resistance to PIs was detected in our study; minor drug resistance to the PIs, A71V/T and L10I, were detected during second-line therapy.

After switching to second line ART, newly acquired NRTIs resistance mutations at position 184, which are associated with resistance to 3TC, were detected in nine HIV/AIDS patients. The M184 V/I mutation was detected in five patients, one patient, two patients, and one patient at the 6, 12, 18, and 48 month timepoints, respectively. Mutation K65R, associated with resistance to TDF, was found in four patients at baseline; however, after switching to second-line ART, K65R mutations were not detected. TAM (M41 L, K70R, L210 W, and T215F) and D67N mutations, associated with resistance to TDF, were detected during second-line ART. NRTI drug-resistance mutations were not found in some patients from the baseline point to 48 months after beginning second-line treatment (NRTIs resistance mutations are shown in Table 3). Mutations at position 103, 106, and 190 are associated with resistance to NNRTIs and were detected during treatment (Table 4).

**Table 3:** NRTIs drug resistance mutations during the second-line antiretroviral treatment

ID	NRTI Mutations at different time-points					
	Baseline	Second-line therapy(months)				
		6	12	18	24	48
1001	M41LM,E44DE,K70KR,L210 W,T215Y	–	–	–	–	K70Q, ***M184I***
1002	None	None	None	None	None	None
1007	M41 L,D67E,K70R,M184I,T215F,K219Q	M41 L,D67E,T215F,K219Q	–	–	–	–
1013	None	–	–	–	None	
1022	D67N,K70KR,L74 V,Q151KLMQ,T215IT,K219Q	D67N,K70R,L74 V,***M184I***, K219Q	D67N,K70R,L74 V, ***M184I***, K219Q	D67N,L74 V, ***M184I***,K219Q	D67N,T69NT,L74 V,***M184I***, K219Q	***M184 V***, K219Q
1026	M41 L,T69D,V118IV, L210 W,T215Y,K219KR	–	–	–	–	None
1027	M41 L,T69ADNT,L74LV,L210 W,T215Y,K219KN	M41 L,T69D, L210 W,T215Y	M41LM,L210LW, T215DY	M41 L,T215Y	T69AT,L210LW, T215D	None
1028	None	None	None	None	None	None
1029	None	***T69NT***	***M41 L,M184 V, L210 W,T215Y***	–	–	–
1031	None	–	–	–	None	None
1032	None	–	–	None	None	–
1036	None	None	None	None		
1045	None	None	None	_	_	None
1049	None	None	None	None		
1056	None	None	None	None	None	–
1062	None	None	None	None		
1067	None	None	–	None	–	–
1068	None			None	None	None
1069	M41 L,E44DE,D67N,L74 V,V118IV,M184 V,L210GW,T215Y	M41 L,D67N,L74 V,M184 V,L210GW,T215Y	–	–	–	–
1076	None	_	_	***T69ST,M184 V***		***M184I***
1078	M184MV,T215FIST	–	–	–	None	–
1083	M41 L,D67NS,T69NT,K70R,T215F,K219E	–	–	–	–	M41 L, D67N, K70R, T215F, K219E
1106	T215FL	–	None	T215FIST	–	–
1126	None	–	–	None	–	None
1134	None	None	None	None	–	–
1135	None	–	None	None	–	–
1136	T215SY	–	–	–	–	T215Y
1155	M41 L,E44DE,D67N,M184I,L210 W,T215Y,K219KR	M41 L,D67N,M184I,L210 W,T215Y	–	–	–	None
1161	None	–	None	–	–	–
1165	None	None	–	–	–	–
1169	M41 L,L210LW,T215FY	M41 L,L210 W,T215Y	L210 W,T215F	M41 L,***M184 V***,L210 W,T215Y	–	None
1170	None	None	None	–	–	–
1173	None	–	–	None	–	–
1174	T215Y	T215Y	T215Y	None	–	–
1175	V75I	–	V75I,***T215Y***	–	–	–
1181	None	None	None	–	–	–
1182	None	–	–	None	–	–
1195	M41LM,L210LW,T215Y	M41 L,***M184 V***,T215Y	–	–	–	–
1196	M41 L,T69P,K70R,L210 W,T215FY,K219E	M41 L,T69PS,K70R,***M184 V***,L210 W,T215Y,K219E	–	–	–	–
1197	M184 V	M41LMV,M184 V, T215Y	–	–	–	–
1198	T215F	M41 L,T215F	–	M41LM,T215FIST	–	None
1199	None	None	–	–	–	–
1202	None	–	None	None	–	None
1203	M41 L,E44D,L210 W,T215Y	M41 L,E44DE,***T69ADNT***,***V118I***,***M184 V***,L210 W,T215Y	–	–	–	–
1205	M41 L,E44D,D67N,K70KR,L210 W,T215Y,K219EK	M41LM,E44DE,D67DN,K70KR,***M184 V,***L210 W,T215Y,K219E	D67N,K70R,***M184 V***,L210 W,T215Y,K219E	M41 L,D67N,K70R,***M184 V***,L210 W,T215Y,K219E	–	–

NRTIs: nucleoside reverse transcriptase inhibitor; “—”: No sequence obtained; None: no drug-resistant mutation was detected; Bold font: newly acquired mutation

**Table 4:** NNRTIs drug resistance mutations during the second-line antiretroviral treatment

ID	NNRTI Mutations at different time-points					
	Baseline	Second-line therapy(months)				
		6	12	18	24	48
1001	V106AV,Y181C,H221Y	–	–	–	–	K103 N
1002	None	None	None	None	None	None
1007	K103 N,Y181C	K103 N,Y181C	–	–	–	–
1013	K103 N	–	–	–	K103 N	
1022	V90I,K103S,G190A	V90I,K103S,G190A	K103S,G190A	K103S,G190A	K103S,G190A	V90I, K103S, G190A
1026	V106A,V108I,F227 L	–	–	–	–	None
1027	V108I,Y181C,H221Y	V108I,Y181C,H221Y	V108I,Y181C	V108I,Y181C	V108I,Y181C	V108I, Y181CY
1028	None	None	None	None	None	None
1029	Y181C,H221HY	V106A,F227 L				
1031	K103 N	–	–	–	K103 N	–
1032	H221Y	–	–	None	None	–
1036	None	V179DV				
1045	K103 N,P225HY,F227FL	K103 N,P225HPSY,F227FL	K103 N,P225HP,F227FL			K103 N
1049	None	None	None	None		
1056	None	None	None	None	None	–
1062	None	None	None	None		
1067	None	None	–	None	–	–
1068	K103KN			None	None	None
1069	K103 N,Y181C,K238H	K103 N,Y181C,K238H	–	–	–	–
1076	None			None	None	None
1078	K101KPQT,K103KN	–	–	–	None	–
1083	Y188L	–	–	–	–	Y188L
1106	V179IL,Y188FHLY	–	None	Y188FHLY	–	–
1112	None	V106IV,G190S				
1126	None	–	–	None	–	–
1134	None	None	None	None		
1135	K103KN	–	K103 N	None	–	–
1136	K103S,G190A,H221HY	–	–	–	–	K103S, G190A
1155	K101E,G190S	K101E,G190S	–	–	–	None
1161	K103 N,Y181C,H221Y	–	K103 N,Y181C	–	–	–
1165	None	None	–	–	–	–
1169	K101HKNQ,K103KN,G190A	K101H,G190A	K101H,G190A	K101H,G190A	–	None
1170	None	None	None	–	–	–
1173	None	–	–	None	–	–
1174	V106I,V108I,G190A	V106I,V108I,G190A	V108I,G190A	None	–	–
1175	K103 N	–	K103 N	–	–	–
1181	None	None	None	–	–	–
1182	G190S	–	–	None	–	–
1195	K103 N,V108I,P225H,K238 T	K103 N,V108I,P225H,K238 T	–	–	–	–
1196	V106A,F227Y,M230 L	V106A,F227Y,M230 L	–	–	–	–
1197	K101HN,V108I,Y181C,H221Y	K101HN,V108I,Y181C,H221Y	–	–	–	–
1198	Y188L	V90I,Y188L		Y188FLY		V106IV
1199	None	–	–	–	–	None
1202	None	–	None	None	–	None
1203	K103 N,Y181C	K103 N,Y181C	–	–	–	–
1205	V106 M,Y188C	V106 M,Y188CFLR	V106 M,Y188L	V106 M,Y188L	–	–

*NNRTIs* Non-nucleoside reverse transcriptase inhibitor; “—”:No sequence obtained; None**:** no drug-resistant mutation was detected;

### Analysis of factors associated with ineffective viral suppression

At least 12 months after switching to the second-line regimen, we identified 31 patients whose viral load had rebounded; 28 of which whose viral load was> 1000 copies/ml. Logistical regression analysis was used to identify factors associated with ineffective viral suppression. The results were as follows: 1) medication adherence (the past month) below 90% (vs. 90–100%) (aOR = 22.74, 95%CI: 3.38–152.59); 2) compliance unknown (vs. 90–100%) (aOR = 9.40, 95%CI: 2.98–29.67), these two factors were protective toward ineffective viral suppression. 3) baseline high level resistance to 3TC/TDF (vs. sensitive) (aOR = 0.10, 95%CI: 0.24–0.43); and 4) baseline low/moderate level resistance to 3TC/TDF (vs sensitive) (aOR = 0.29, 95%CI: 0.10–0.83) (Table 5), the two factors were protective toward effective viral suppression.

**Table 5:** Univariate and multivariate analyzes the factors associated with ineffective viral suppression

Predictor		OR(95%CI)	*p*	a OR(95%CI)	*p*
Age(years)	≤45	1			
	> 45	0.72(0.35,1.48)	0.367		
Gender	Male	1			
	Female	0.74(0.36,1.55)	0.431		
Education degree	illiteracy	1			
	Primary school	1.23(0.44,3.44)	0.699		
	Junior middle school	0.91(0.30,2.72)	0.865		
Baseline CD4 + T cells/μl	CD4 ≤ 50	2.86(0.64,12.7)	0.168	2.68(0.35,20.35)	0.340
	50 < CD4 ≤ 200	1.63(0.77,3,47)	0.202	1.20(0.45,3.19)	0.450
	CD4 > 200	1			
Baseline viral load copies/ml	1000 < HIV RNA ≤ 10,000	1		1	
	10,000 < HIV RNA ≤ 10,000	1.31(0.52,3.31)	0.567	1.07(0.34,3.32)	0.914
	HIV RNA > 100,000	3.46(1.27,9.37)	0.015	2.18(0.63,7.61)	0.221
Duration of first-line therapy (years)	≤4	1			
	> 4	1.35(0.60,3.04)	0.475		
Baseline 3TC/TDF resistance	sensitive	1		1	
	low/moderate resistance	0.28(0.12,0.66)	0.004	0.29(0.10,0.83)	0.022
	high resistance	0.16(0.05,0.49)	0.001	0.10(0.24,0.43)	0.002
medication adherence	90–100%	1		1	
	< 90%	9.98(2.00,49.89)	0.005	22.74(3.38,152.59)	0.001
	unknown	7.48(2.95,18.97)	< 0.001	9.40(2.98,29.67)	< 0.001

*3TC* Lamivudine, *TDF* Tenofovir

## Discussion

In our study, patients were recruited from Henan province in China; Henan was one of the earliest areas to begin free first-line ART for HIV-infected citizens and was the first to initiate second-line antiretroviral therapy as described in the National Free Antiretroviral Treatment Program (NFATP). During the 4 years of follow-up, patient CD4^+^ T cell counts were significantly higher than at baseline and viral load was significantly lower. The median CD4^+^ T counts increased by an average of 146 cells/L and 70% (63/90) of patients achieved viral suppression within 24 months in our study, which was similar to results reported from South Africa in which CD4^+^ T counts increased by an average of 177cells/L and 75% (74/99) of patients achieved a viral load < 1000 copies/mL within 24 months of beginning second line therapy [27]. We also analyzed the efficacy of 48 months of second-line therapy; CD4^+^ T counts increased to 189 cells/L and 75.3% (64/85) of patients achieved a viral load < 1000 copies/mL. These results indicate that long-term treatment was effective in our cohort.

Drug-resistance mutations were detected when the viral load reached > 1000 copies/ml during second-line ART follow-up; yet, our results show that newly acquired NRTI resistance mutations were few. The M184 V/I mutation was detected in nine patients after switching to second-line therapy, which can attributed to the use of 3TC in the program. Boyd et al. found that one patient newly acquired M184 V resistance mutations after 96 weeks second-line ART [22]. TDF was used in the second-line program; the mutations associated with resistance to TDF were K65R, TAM (M41 L, K70R, L210 W, T215F), D67N, K70E, and Y115F. The K65R mutation causes intermediate/high-level resistance to TDF; use of TAMs (M41 L, K70R, L210 W, T215F) can reduce TDF susceptibility and cause intermediate/low level resistance to TDF. The D67N mutation, present with other TAMs, can result in reduced susceptibility to TDF. The K70E and Y115F mutations cause low-level resistance to TDF as well [28, 29]. In our study, K65R was detected in four patients at baseline, which may be related to drugs used in the first-line ART; these four patients achieved viral suppression during second-line treatment. However, K65R was not detected in other patients after switching to second-line ART, which was consistent with Boyd et al. [22]. TAM resistance mutations did not accumulate significantly in our study, but the results from Reynolds et al. show that TAM (M41 L, L210 W, and T215F/Y) and M184I/V mutations related to NRTI drug resistance increased after patients switched to the second-line regimen [19]. These inconsistent results may be due to different first-line treatment regimens, different HIV subtypes, or the differences in patients’ drug metabolism or medication adherence.

Major PI resistance mutations were not detected using the “in-house” genotype drug resistance test. However, we obtained HIV Pol sequences using monoclonal methods; the results of a rootless radial tree generated via phylogenetic analysis at the two time points (baseline and after 48 months of second-line treatment) showed significant evidence of viral evolution, suggesting that resistance surveillance should be continued.

After switching to the second-line regimen, 31 patients exhibited increased viral load; of these, 28 patients exhibited viral loads > 1000 copies/ml after at least 12 months of second-line therapy. We performed a logistical regression analysis of the data; our results indicated that medication adherence (in the past month) below 90%, compliance or when medication adherence was unknown was associated with ineffective viral suppression. These results are consistent with the work reported by Murphy et al. [27]. In a study conducted in India, Khan et al. reported 62% of patients with failure of second-line therapy had a subsequently undetectable viral load after a median duration of 3 months if they remained on second-line ART after enhanced adherence support [30]. Taken together, these data suggest that more effective measures are needed to improve patient medication adherence and thus improve utilization of the limited medical resources.

In our cohort, baseline resistance to 3TC/TDF resulted from ineffectiveness of the second-line ART; which was similar to the research reported regarding second line treatment in Africa [15, 31]. Paton et al. also found that greater sensitivity to NRTI was associated with poorer viral load suppression; they suggest that NRTI activity in protease inhibitor-based second-line ART was not accurately predicted by the results of genotypic resistance [17]. Some reports indicate that the NRTI resistance mutation, M184 V, can enhance viral sensitivity to TDF [32], which could result in effective virus suppression. We suspect mutations in the reverse transcriptase inhibitor and protease inhibitor regions act synergistically to enhance the virus sensitivity to the PIs; however, this will require further verification. Some researchers have other opinions, as Hosseinipour et al. suggested that first-line treatment failure in patients with wild HIV strains indicates poor medication adherence [15]. Therefore, we also need to compare the second-line treatment results between the baseline NRTI-resistant and baseline NRTI-sensitive patients under conditions of good medication adherence in future.

## Conclusions

Long-term second-line antiviral therapy in the Henan region was effective. During the four years of second-line therapy, drug mutations conferring resistance to NRTIs were observed among patients receiving second-line ART, although major drug resistance mutations were not detected in the PI region. Drug resistance monitoring should be continued to prevent the spread of resistant strains. In addition, medication adherence supervision and management should be increased in order to improve the effectiveness of antiviral drugs.

## Abbreviations

3TC: Lamivudine
AIDS: Acquired immunodeficiency syndrome
ART: Anti-retroviral therapy
HIV: Human immunodeficiency virus
Lpv/r: Lopinavir/ritonavir
NNRTI: Non-nucleotide reverse transcriptase inhibitor
NRTI: Nucleotide reverse transcriptase inhibitor
PI: Protease inhibitor
TAM: thymidine analogue mutation
TDF: Tenofovir
